# Supplementary material for: Sequential Chromogenic IHC: Spatial Analysis of Lymph Nodes Identifies Contact Interactions between Plasmacytoid Dendritic Cells and Plasmablasts
Source: Cancer Res Commun. 2023 Jul 13;3(7):1237–47. doi: 10.1158/2767-9764.CRC-23-0102 (PMC10361537; doi:10.1158/2767-9764.CRC-23-0102)
Supplement: Supplementary Tables S1-S4 — Table S1: Patient characteristics (n=10) Table S2: Sequential IHC panel information Table S3: Secondary HRP Round Considerations Table S4: Comprehensive database information for mIHC panel development [file crc-23-0102-s02.docx]

Table S1**:** Patient characteristics (n=10)

| **Characteristic:** | **N(%)**  **Overall** |
| --- | --- |
| Age at diagnosis | 58.6 (14.15) * |
| Female | 3 |
| Male | 7 |
| Race: White/Caucasian | 10 |
| Race: African American | 0 |
| **Overall Anatomic Stage at dx:** |  |
| Stage-1 | 0 |
| Stage-2 | 1 |
| Stage-3 | 1 |
| Stage-4 | 8 |
| **Tumor location:** |  |
| Neck | 3 |
| Tongue/Maxilla | 4 |
| Larynx/Pharynx/Tonsil | 3 |
| **Nodal status** |  |
| 0 (no nodal metastasis) | 1 |
| 1 (metastases in 1-3 nodes) | 3 |
| 2 (metastases in ≥4 nodes) | 6 |
| **Treatment type:** |  |
| Surgery alone | 3 |
| Surgery + radiation | 5 |
| Surgery + CRT | 2 |

*Average (SD)

Table S2, Sequential IHC panel information.

| **Marker** | **Host Species** | **Dilution** | **Reaction** | **Clone** | **Vendor** | **Cell type** |
| --- | --- | --- | --- | --- | --- | --- |
| CD56 | Rabbit IgG | 1:500 | Overnight 4°C | MA1-06801 | Novus Biologicals | NK cells |
| CD20 | Goat IgG | 1:500 | Room temp, 30 min | polyclonal | Abcam | B cells |
| CD66b | Mouse IgG | 1:600 | Room temp, 30 min | G10F5 | BD Biosciences | Granulocytes |
| PD1 | Goat IgG | 1:200 | Overnight 4°C | polyclonal | R&D Systems | T follicular helper |
| IgM | Mouse IgG | 1:200 | Overnight 4°C | IM260 | Novus Biologicals | Multiple |
| CD3 | Rat IgG | 1:250 | Room temp, 30 min | CD3-12 | Abcam | T cells |
| IgE | Goat IgG | 1:1000 | Room temp, 30 min | polyclonal | Novus Biologicals | Multiple |
| HLA-DR/DP/DQ | Mouse IgG | 1:500 | Room temp, 30 min | WR18 | LSBio | Multiple |
| CD169 | Sheep IgG | 1:200 | Room temp, 30 min | polyclonal | R&D Systems | Sinusoidal macrophages |
| CD11c | Rabbit IgG | 1:200 | Room temp, 30 min | SI19-06 | Novus Biologicals | Dendritic cells |
| IgA | Goat IgG | 1:10,000 | Overnight 4°C | polyclonal | Novus Biologicals | Multiple |
| IgG1 | Rabbit IgG | 1:500 | Room temp, 30 min | RM117 | Novus Biologicals | Multiple |
| BDCA2 | Goat IgG | 1:100 | Room temp, 1 hr | polyclonal | Novus Biologicals | Plasmacytoid dendritic cells |
| CD138 | Mouse IgG | 1:50 | Room temp, 30 min | MI15 | ThermoFisher | Plasmablasts and plasmacells |
| CD38 | Mouse IgG | 1:20 | Room temp, 1 hr | 38C03 (SP32) | ThermoFisher | Plasmacells |
| CD88 | Rabbit IgG | 1:500 | Overnight 4°C | polyclonal | Novus Biologicals | Recirculating monocytes |
| CD141 | Sheep IgG | 1:20 | Room temp, 2 hr | polyclonal | Novus Biologicals | Type-1 conventional DCs |
| CD1c | Mouse IgG | 1:200 | Room temp, 1 hr | OTI2F4 | Novus Biologicals | Type-2 conventional DCs |
| IgG3 | Rabbit IgG | 1:500 | Overnight 4°C | RM119 | Novus Biologicals | Multiple |
| PanCK | Rabbit IgG | 1:500 | Room temp, 1 hr | polyclonal | Novus Biologicals | Squamous carcinoma cells |
| CD4 | Mouse IgG | 1:150 | Room temp, 30 min | OT12H8 | LSBio | CD4 T cells |
| FDC | Mouse IgG | 1:1000 | Overnight 4°C | CNA.42 | Novus Biologicals | Follicular dendritic cells |
| Ki67 | Rabbit IgG | 1:500 | Room temp, 30 min | SP6 | MilliporeSigma | Proliferation |
| CD8 | Mouse IgG | 1:50 | Room temp, 30 min | C8/144B | ThermoFisher | CD8 T cells |
| Tbet | Rabbit IgG | 1:200 | Room temp, 1 hr | D6N8B | Cell Signaling | Activation |
| CD68 | Mouse IgG | 1:2000 | Room temp, 30 min | KP1 | Novus Biologicals | Macrophages |
| BCL6 | Rabbit IgG | 1:50 | Room temp, 1 hr | polyclonal | Novus Biologicals | Multiple |

Table S3. Secondary HRP Round Considerations.

| **Primary Antibody Species** | **Secondary HRP Polymer** | **Manufacturer** | **Round Considerations** |
| --- | --- | --- | --- |
| Rabbit | Horse αRabbit | Vector Labs | For cycles that have goat derived antibodies |
| Rabbit | Goat αRabbit | Nacalai USA | For cycles that do not have goat derived antibodies* |
| Goat | Rabbit αGoat | Nacalai USA | Place goat antibody before mouse and rat derived antibodies |
| Mouse | Goat αMouse | Nacalai USA | Use in any round, but after goat derived antibodies |
| Rat | Goat αRat | Nacalai USA | Use in any round, but after goat derived antibodies |
| Sheep | Donkey αSheep | R&D Systems | Use in any round |

*Due to cost consideration of Horse αRabbit HRP polymer at the time of experimentation.

Table S4. Comprehensive database information for mIHC panel development.

| **Database Column** | **Information captured** |
| --- | --- |
| Marker | What the antibody detects |
| Species | Species the antibody was produced in: Mouse, Rabbit, Rat, Goat, Sheep |
| Reactivity | Species the antibody reacts to: Human |
| Dilution | Specific dilution the antibody was tested |
| Incubation | 30 minutes – 2 hours a room temperature, overnight at 4°C |
| AEC incubation | The amount of time the slides was incubated with visualization buffer (this was used to inform planning and timing of protocol) |
| Protein Block incubation | Amount of time protein block was incubated (at room temperature) |
| Antigen Retrieval | Citrate (pH 6.0) or Tris-EDTA (pH 9.0) |
| Slide ID | Unique identifier of slide |
| Cycle | The specific cycle number the slide was tested: C01, C02, etc. |
| Number of Rounds | The cumulative number of rounds a unique slide has been subjected to irrespective of cycle. |
| Worked | Yes, No, Yes with background, Yes with faint staining, Background only, No with carryover |
| Tissue Type | Specific type of tissue: Lymph node, tonsil, skin |
| Experiment | The name of the experiment and data location (to access images) |
| Clone | Specific clone of antibody |
| Company | Manufacturer of antibody |
| Catalog Number | Catalog number |
