# Supplementary material for: Sequential Chromogenic IHC: Spatial Analysis of Lymph Nodes Identifies Contact Interactions between Plasmacytoid Dendritic Cells and Plasmablasts
Source: Cancer Res Commun. 2023 Jul 13;3(7):1237–47. doi: 10.1158/2767-9764.CRC-23-0102 (PMC10361537; doi:10.1158/2767-9764.CRC-23-0102)

Figure S1. Distortion maps. In order to align all the biomarker images for a given sample, each image needs to be corrected for distortions that happen during the staining procedure. This step aims to rectify micro- and macro-anatomical shifts in the tissue sections. Two examples of the calculated shifts (dx, dy and amplitude) and support points for CD3 and CD20.

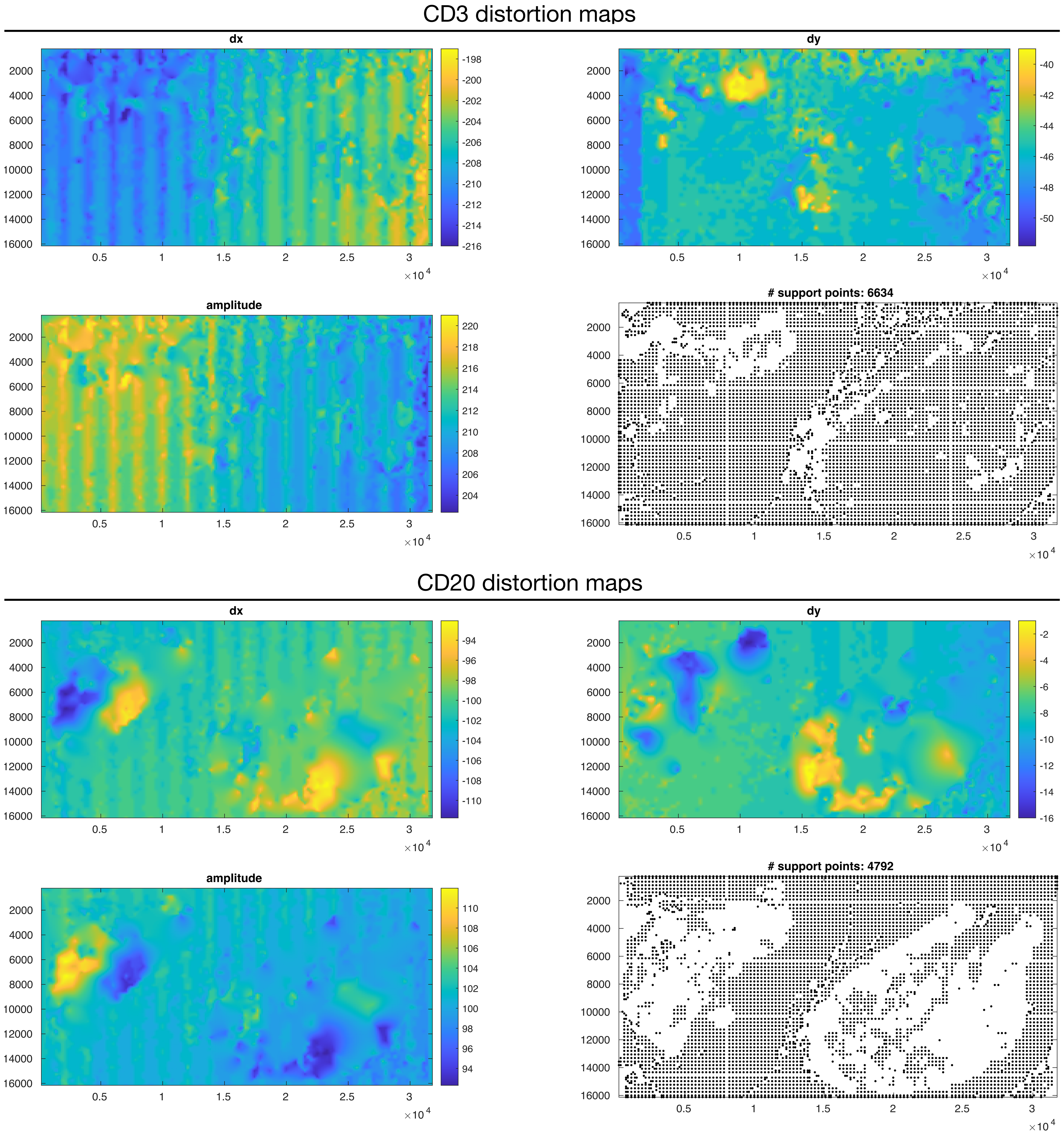

Supplement: Supplementary Figure S1 — Distortion maps. In order to align all the biomarker images for a given sample, each image needs to be corrected for distortions that happen during the staining procedure. This step aims to rectify micro- and macro-anatomical shifts in the tissue sections. Two examples of the calculated shifts (dx, dy and amplitude) and support points for CD3 and CD20. [file crc-23-0102-s03.pdf]
