# Supplementary material for: Sequential Chromogenic IHC: Spatial Analysis of Lymph Nodes Identifies Contact Interactions between Plasmacytoid Dendritic Cells and Plasmablasts
Source: Cancer Res Commun. 2023 Jul 13;3(7):1237–47. doi: 10.1158/2767-9764.CRC-23-0102 (PMC10361537; doi:10.1158/2767-9764.CRC-23-0102)
Supplement: Supplementary Figure S2 — Alignment results. Comparison of before and after alignment for CD3 (red, top) and CD20 (red, bottom) superimposed with hematoxylin (green). [file crc-23-0102-s04.pdf]

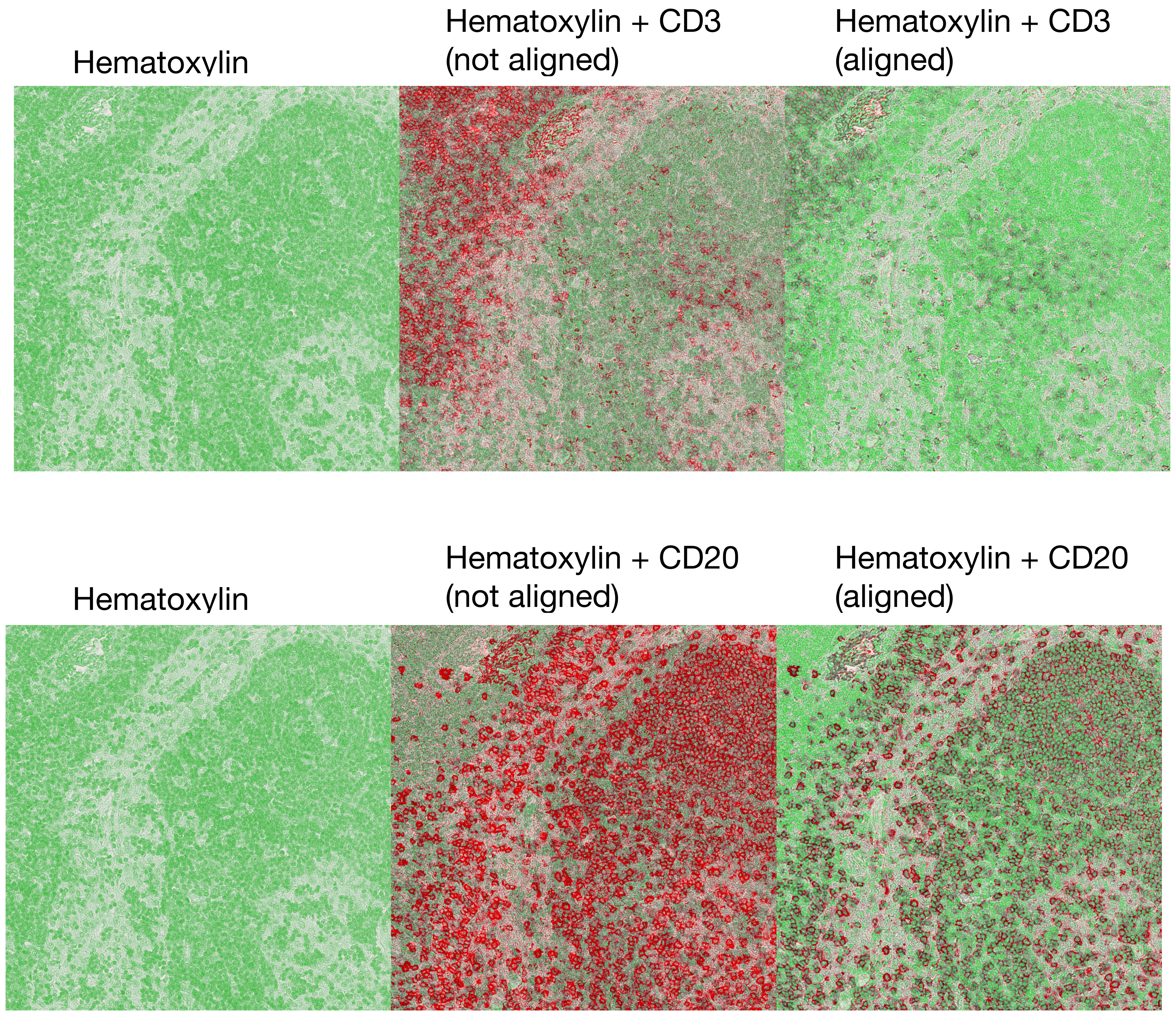

Figure S2. Alignment results. Comparison of before and after alignment for CD3 (red, top) and CD20 (red, bottom) superimposed with hematoxylin (green).
