# Supplementary material for: Sequential Chromogenic IHC: Spatial Analysis of Lymph Nodes Identifies Contact Interactions between Plasmacytoid Dendritic Cells and Plasmablasts
Source: Cancer Res Commun. 2023 Jul 13;3(7):1237–47. doi: 10.1158/2767-9764.CRC-23-0102 (PMC10361537; doi:10.1158/2767-9764.CRC-23-0102)
Supplement: Supplementary Figure S3 — Trained networks detected artifacts and tissue of interest limits, and excluded them from the analysis. [file crc-23-0102-s05.pdf]

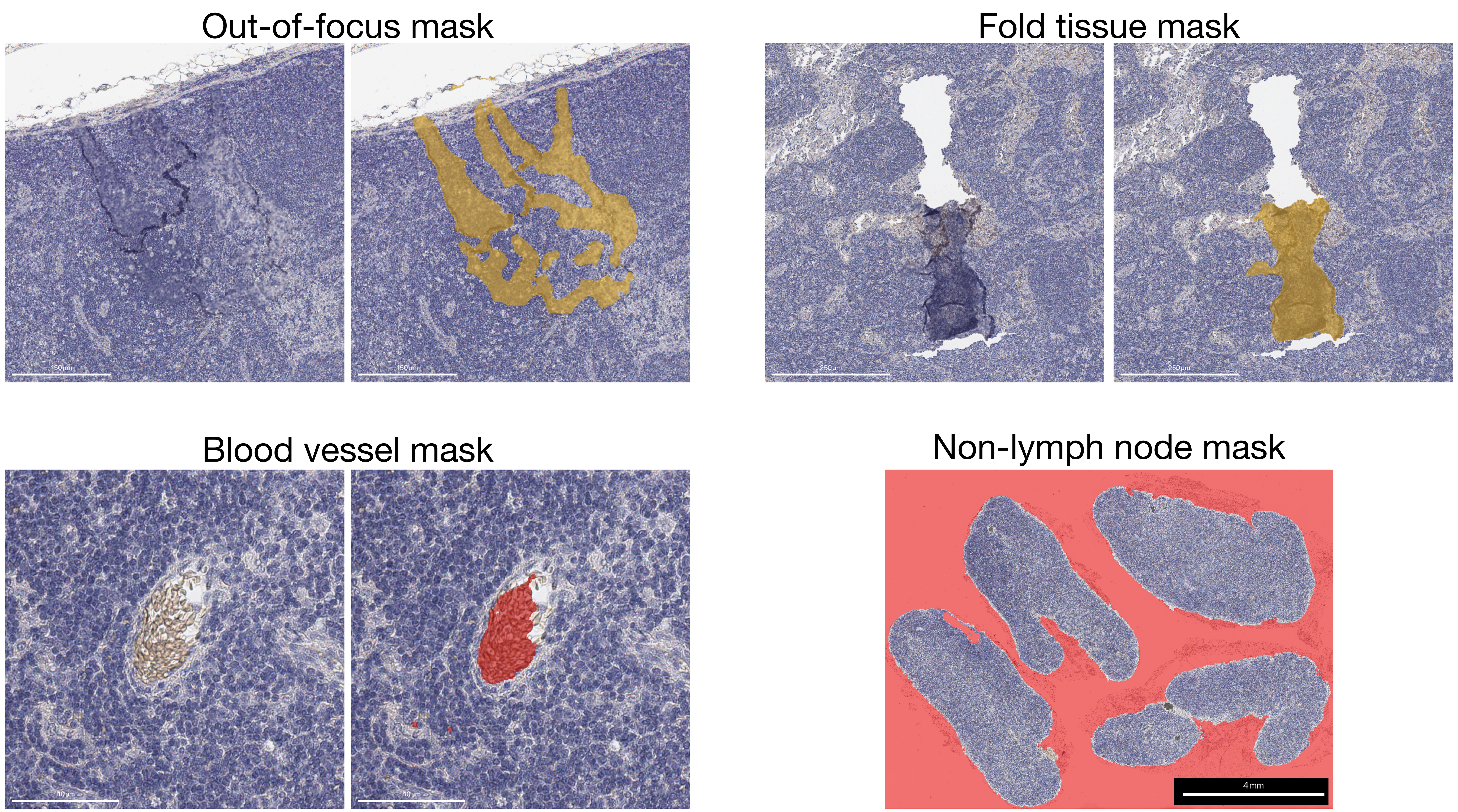


Figure S3. Trained networks detected artifacts and tissue of interest limits, and excluded them from the analysis.
