# Supplementary material for: Sequential Chromogenic IHC: Spatial Analysis of Lymph Nodes Identifies Contact Interactions between Plasmacytoid Dendritic Cells and Plasmablasts
Source: Cancer Res Commun. 2023 Jul 13;3(7):1237–47. doi: 10.1158/2767-9764.CRC-23-0102 (PMC10361537; doi:10.1158/2767-9764.CRC-23-0102)
Supplement: Supplementary Figure S4 — Avoid cross-reactivity of secondary reagents. Using a horse anti-rabbit (see Figure 5) to detect IgG1 (A) or CD11c (B) in round 1 does not lead to cross reactivity with the rabbit anti-goat secondary reagent used in the next round to detect BDCA2 and IgA, respectively. [file crc-23-0102-s06.pdf]

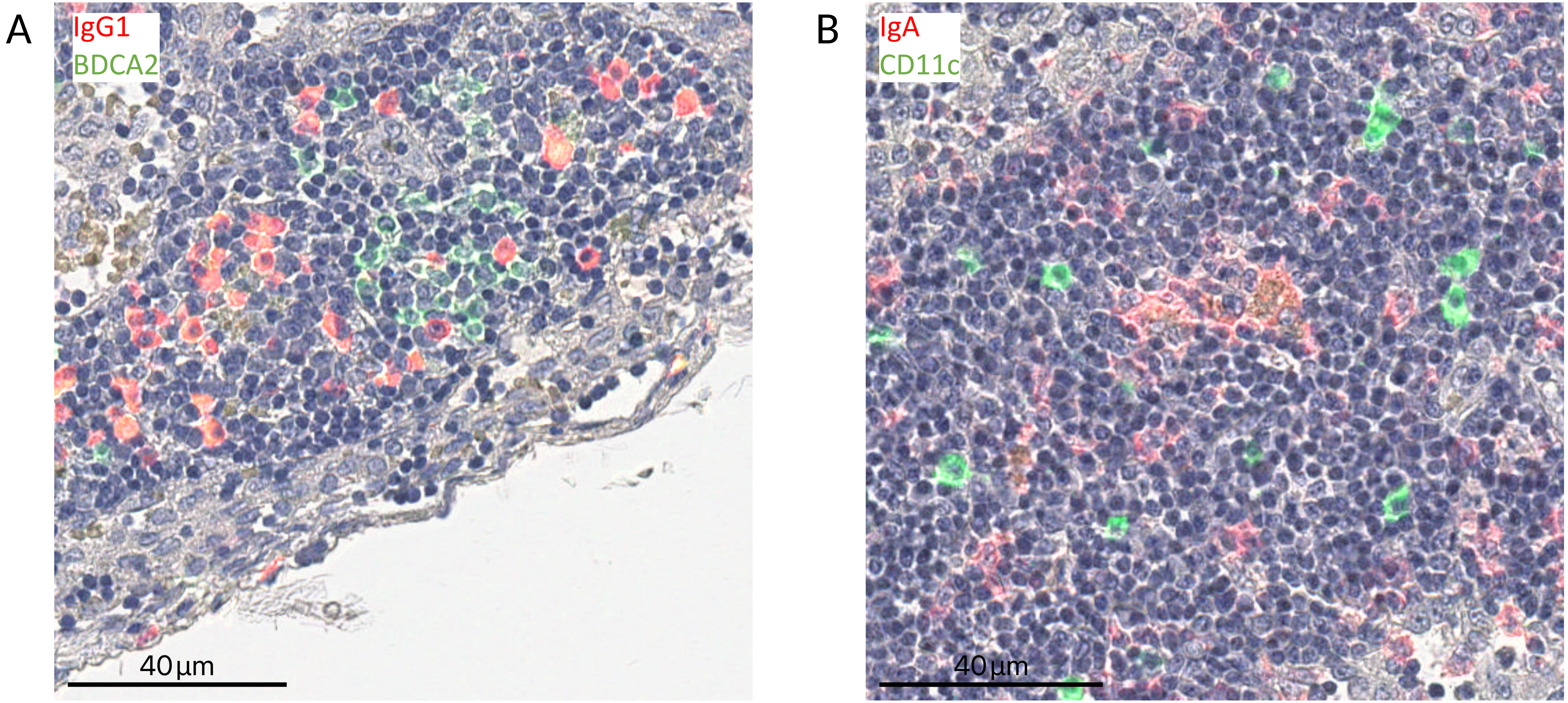

Figure S4. Avoid cross-reactivity of secondary reagents. Using a horse anti-rabbit (see Figure 5) to detect IgG1 (A) or CD11c (B) in round 1 does not lead to cross reactivity with the rabbit anti-goat secondary reagent used in the next round to detect BDCA2 and IgA, respectively.
